# Miniaturized wireless gastric pacing via inductive power transfer with non-invasive monitoring using cutaneous Electrogastrography

**DOI:** 10.1186/s42234-021-00074-8

**Published:** 2021-08-24

**Authors:** Andrew Perley, Mehrdad Roustaei, Marcelo Aguilar-Rivera, David C. Kunkel, Tzung K. Hsiai, Todd P. Coleman, Parinaz Abiri

**Affiliations:** 1 Department of Bioengineering, University of California, San Diego, La Jolla, CA 92093 USA; 2 Department of Bioengineering, University of California, Los Angeles, Los Angeles, CA 90095 USA; 3 Division of Gastroenterology, University of California, San Diego, La Jolla, CA 92093 USA; 4 Department of Medicine, University of California, Los Angeles, Los Angeles, CA 90095 USA

**Keywords:** Gastric pacing, Gastric pacemaker, Wireless electrical stimulation, Wireless pacing, Electrogastrogram, Electrogastrography, Gastroparesis

## Abstract

**Background:**

Gastroparesis is a debilitating disease that is often refractory to pharmacotherapy. While gastric electrical stimulation has been studied as a potential treatment, current devices are limited by surgical complications and an incomplete understanding of the mechanism by which electrical stimulation affects physiology.

**Methods:**

A leadless inductively-powered pacemaker was implanted on the gastric serosa in an anesthetized pig. Wireless pacing was performed at transmitter-to-receiver distances up to 20 mm, frequency of 0.05 Hz, and pulse width of 400 ms. Electrogastrogram (EGG) recordings using cutaneous and serosal electrode arrays were analyzed to compute spectral and spatial statistical parameters associated with the slow wave.

**Results:**

Our data demonstrated evident change in EGG signal patterns upon initiation of pacing. A buffer period was noted before a pattern of entrainment appeared with consistent and low variability in slow wave direction. A spectral power increase in the EGG frequency band during entrainment also suggested that pacing increased strength of the slow wave.

**Conclusion:**

Our preliminary in vivo study using wireless pacing and concurrent EGG recording established the foundations for a minimally invasive approach to understand and optimize the effect of pacing on gastric motor activity as a means to treat conditions of gastric dysmotility.

## Introduction

For the past two decades, the use of electrical stimulation (ES) as a means to regulate gastric motility has been studied in various models. Most prominently, ES has been investigated as a therapeutic device for refractory gastroparesis, a chronic and debilitating disease that causes severe nausea, vomiting, abdominal fullness, and pain despite the absence of any mechanical blockage of the stomach (Angeli et al. 2015; Nilsson 1996). Various studies have shown long-term reduction of symptoms and improvement in enteral feeding following stimulation of the smooth muscle of the stomach (Abell et al. 2002; McCallum et al. 2011; McCallum et al. 2010; Forster et al. 2001; Lin et al. 2004; Mason et al. 2005; Levinthal and Bielefeldt 2017). While the mechanism behind this symptomatic improvement remains to be fully characterized, gastric electrical stimulation continues to be utilized for the treatment of gastroparesis in refractory cases (Enterra 3116 Implant Manual 2015).

Notably, clinical studies performed thus far have utilized lead-based stimulation devices, functioning similar to a cardiac pacemaker repurposed for gastric stimulation. However, lead-based implants can result in a number of life-threatening complications, including perforation through the stomach wall at the lead electrode insertion site, bowel obstruction and necrosis due to lead entanglement and erosion, and intra-abdominal infection (Enterra 3116 Implant Manual 2015). These challenges are compounded by a missing link between the effect of electrical stimulation in symptom relief and its cause. Recent work has attempted to establish this connection by using concurrent multi-electrode serosal recordings to provide feedback in response to a stimulus (Alighaleh et al. 2021). However, the system’s size (5 × 5 × 1 cm) and the need for invasive deployment of the recording electrodes and stimulation system may lead to post-surgical complications. Attempts have also been made to create a wireless electrical stimulation system to overcome invasive deployment (Rao et al. 2014), but there continue to be challenges in the implant fixation mechanism due to large device size, and safety concerns due to tissue energy absorption in the setting of high-power wireless transmission. The limitations facing a gastric pacer have, therefore, slowed its progression as a potentially powerful tool against gastric dysmotility.

We thereby present a minimally invasive miniature wireless gastric pacemaker to reduce lead-based complications and allow for the advancement of gastric pacing as a potential therapeutic. Our system architecture allowed for long-range wireless power transfer as well as a vascular fixation mechanism to reduce the number of complications associated with surgery, entanglement, and infection. We investigated in vivo gastric pacing on the swine gut, demonstrating the effect of low frequency electrical stimulation on the motor activity of the stomach smooth muscle. We also established that concurrent cutaneous multi-electrode electrogastrogram (EGG) monitoring allows for adequate tracking of slow wave propagation patterns by comparing spatial patterns to those captured from serosal electrodes. Our cutaneous EGG-based analysis of the stomach motor activity not only enabled a non-invasive feedback system for therapy, but also established a basis for the mechanism by which gastric pacing can be achieved through optimized stimulus delivery as means to treat conditions of gastric dysmotility.

## Materials & methods

### Pacemaker system design

**Fig. 1:**
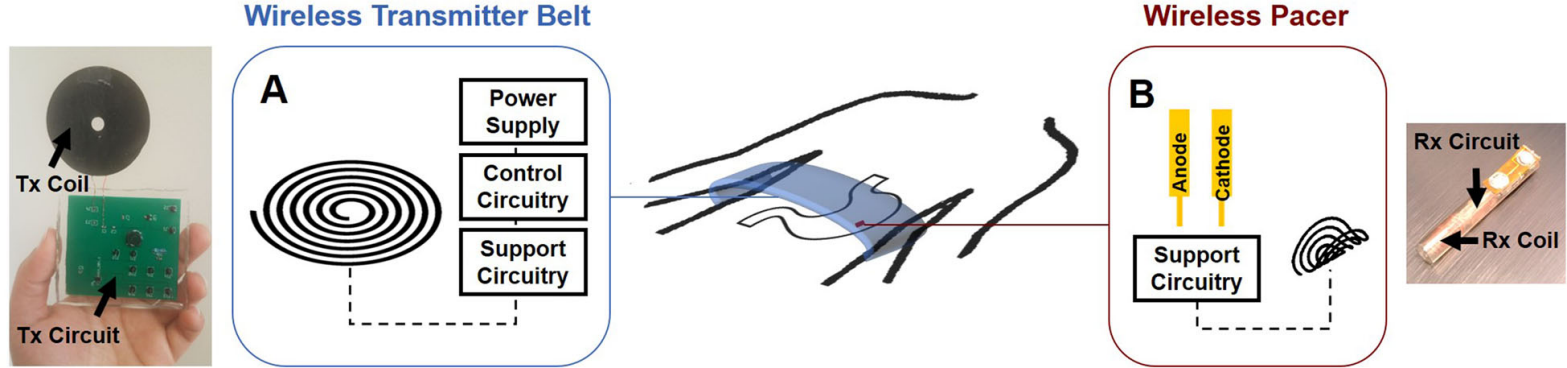
Gastric Pacer System Design. **A** Wireless power transmitter (Tx) consisted of a 26 AWG copper wire, shaped into a planar circular coil 40 mm in diameter, a power supply, support circuitry with a power amplifier and tank circuit, and control circuitry to regulate pacing frequency, duration, and amplitude. **B** Wireless pacer consisted of a 30 AWG copper wire, folded into a semi-cylindrical power-receiving (Rx) coil 2.8 mm in diameter and 15 mm in length, a pair of platinum treated copper electrodes, and a support circuity with a tank circuit and AC-DC converter. For insulation against the conductive blood, the internal components were encapsulated by polydimethylsiloxane. The transmitter would ultimately take the form factor of an external belt to control the intravascular implanted gastric pacer on the stomach serosal wall

We designed a miniature pacing system with a remote-controlled stimulation mechanism to reduce implant size, minimize specific absorption rate (SAR), and improve power transfer efficiency through intermittent transmission, whereby the control circuitry is placed entirely in the wireless transmitter (Fig. 1) (Abiri et al. 2017). Coil design for optimal power transfer efficiency in the setting of restricted venous geometry has been extensively discussed in our previous work (Abiri et al. 2017; Abiri et al. 2020); and while the presented studies were performed via surgical fixation, the device was designed for minimally invasive intravascular deployment similar to a stent. This proposed fixation mechanism has the potential to eliminate the risk of lead dislodgement, entanglement, and perforation through the elimination of the leads connecting the pulse generator to the pacing electrodes. Additionally, the reduction of tissue contact surface area via a smaller implant size can minimize infection rate and mortality. Furthermore, by utilizing a remote-controlled wireless stimulation mechanism, device programming can be performed entirely independent of the implanted pacer using an externally placed wearable belt.

### Electrogastrogram system design

**Fig. 2:**
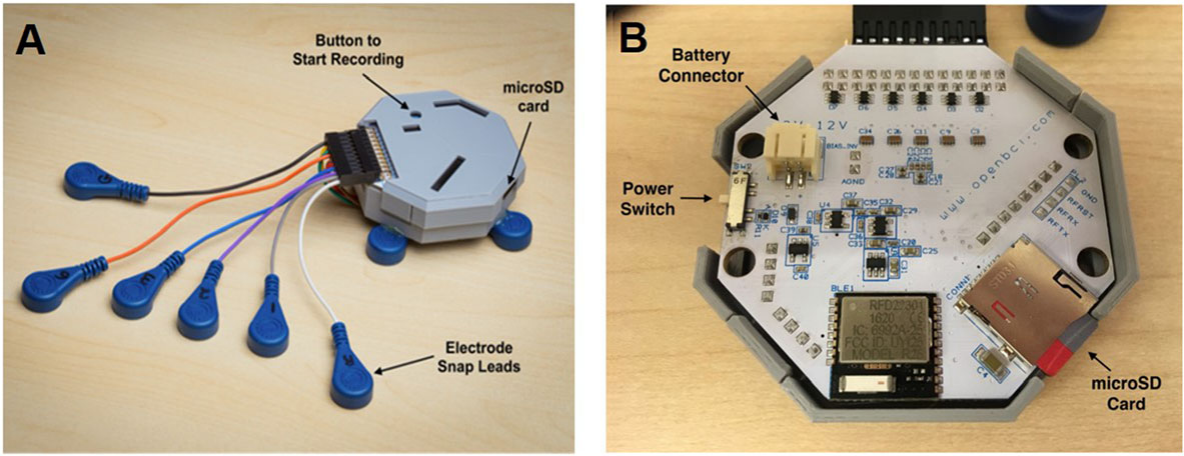
Electrogastrogram System Design. **A** Cutaneous electrode attached to (**B**) 24-bit OpenBCI Cyton board

An 8-channel ambulatory electrogastrogram system was designed using a 24-bit OpenBCI Cyton board that we have previously used in EGG studies (Gharibans et al. 2018) (Fig. 2). This system was designed as an inexpensive and portable solution to recording EGG signals in patients in and out of the clinic. For this experiment, standard 3 M ECG electrodes were used and EGG data was collected at a native sampling frequency of 250 Hz.

### In vivo pacing of swine gut

We performed in vivo pacing along the greater curvature of the body of the stomach in one male Yorkshire pig. The study was approved by the UCLA Office of Animal Research in compliance with the UCLA IACUC protocols. The pig was anesthetized with intramuscular ketamine and midazolam. An abdominal incision was made for access to the stomach.

**Fig. 3:**
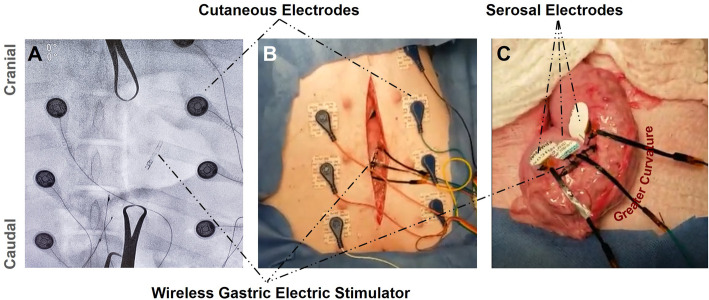
Swine Study Design. **A** Radiographic image of the swine abdomen with implanted gastric pacer and cutaneous EGG recording electrodes demonstrates minimal device footprint upon implantation to reduce potential complications. **B** Photo demonstrating surgical incision site and relative position of the pacer from the skin surface where the pacer transmitter was positioned during stimulation. **C** The segment of the stomach where the wireless pacer and serosal recording electrodes were sutured demonstrate device position along the greater curvature

Five recording electrodes, one reference electrode, and one ground electrode were placed cutaneously on the ventral side around the abdominal incision (Fig. 3A-B). The pacer was sutured on the serosal side of the stomach, with the cathode positioned in the caudal direction and anode positioned in the cranial direction (Fig. 3A-C). Three recording electrodes were sutured around the pacer on the serosal side of the stomach (Fig. 3C). EGG recordings were started approximately 20 min prior to stimulation. Pacing was then initiated according to natural slow wave activity, at a frequency of 0.05 Hz, pulse width of 400 ms, positioned at a wireless range of 10–20 mm to represent surface-to-implant distance. Prior studies have demonstrated a voltage of 6–10 V at this distance range, corresponding to a current range of 6–10 mA in the setting of 1000 Ω impedance (Abiri et al. 2020). Based on this stimulation criteria and prior studies (Abiri et al. 2021), our wireless pacer functioned at a SAR level of 0.6 W/kg at its maximum point, which is less than half the FCC safety threshold. After approximately 40 min of pacing, the stimulus was terminated and EGG recordings were continued for about 5 min following termination.

### Data analysis

The 8 electrophysiologic recordings were collected at a sampling frequency of 250 Hz. All signal processing was done in Python using NumPy and SciPy packages (Harris et al. 2020; Virtanen et al. 2020). Three of these channels were placed directly on the serosal surface of the stomach to directly assess the effect of pacing and to serve as a reference point to test the viability of extracting spatial patterns from non-invasive EGG. The remaining five electrodes were placed cutaneously on the abdomen overlying the stomach. Motion artifacts were rejected using an adaptive Wiener filter as demonstrated in Gharibans et al. (Gharibans et al. 2018). Since the EGG signal has a dominant frequency of 0.05 Hz, the data was decimated to a 1 Hz sampling rate and then further bandpass filtered to a band of 0.03–0.07 Hz using a 4th order zero-phase Butterworth filter to isolate the EGG signal. We applied the Hilbert transform to construct the analytic signal:

1 $$ z(t)=x(t)+i\ast H\left\{x(t)\right\}=a(t){e}^{i\phi (t)}, $$

where *z*(*t*) is the analytic signal, and *a*(*t*) and *ϕ*(*t*) are known as the instantaneous amplitude and phase of the analytic signal.

For any waveform, by taking the angle of the analytic function in the complex plane, we extract the phase *ϕ*(*t*). We express the phase of an electrode at location (*x*_*i*_, *y*_*i*_) and at time t as *ϕ*_*i*_(*x*_*i*_, *y*_*i*_, *t*). In order to compute the direction of slow wave propagation at time, we computed:

2 $$ direction(t)= angle\left(\overline{\nabla {\phi}_i(t)}\right), $$

where $\overline{\nabla {\phi}_i(t)}$ is the average phase gradient over all electrodes. The direction of the slow wave provides information regarding the average relative angle of the slow wave propagation at any time, t, with respect to a Cartesian coordinate system of choice.

In order to determine the extent to which the array of phase values constitute a spatial plane wave propagating, we computed the phase gradient directionality (PGD), which takes a value on [0,1], and is calculated as:

3 $$ PGD(t)=\frac{\left\Vert\ \overline{\phi (t)}\ \right\Vert }{\overline{\mid \left|\phi (t)\right|\mid }}. $$

As PGD increases, the spatial phase relationships more closely represent plane wave propagation. For the remainder of the analyses, we calculated directional statistics only at times when PGD was > 0.5 to reduce false discovery rate as we have done in previous analyses (Gharibans et al. 2017). The EGG power as a function of time was also plotted to demonstrate the frequency response of the slow wave to pacing. A periodogram of the EGG signal with a 256-point window was constructed using a standard short-time Fourier Transform. To extract the power in the EGG signal over time, the highest average power signal in the [0.45, 0.55] Hz frequency band was chosen as the EGG signal.

## Results & discussion

**Fig. 4:**
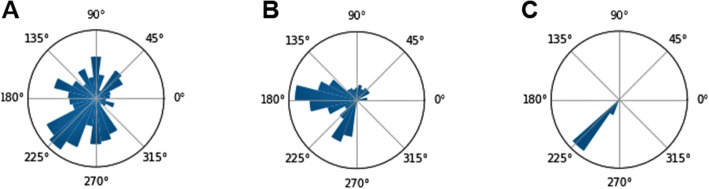
Pacing Impact on the Slow Wave. Slow wave spatial histogram (**A**) prior to pacing, **B** during pacing before entrainment period, and **C** during pacing and during entrainment period, demonstrate the effect of gastric pacing on motor activity over time. Swine stomach under anesthesia is thus shown to convert from dysregulation to uniform activity once pacing induced motor entrainment

Data recording included an approximately 20-min period before, 40-min period during, and 5-min period after artificial pacing. Prior to the initiation of pacing, the slow wave propagation demonstrated distributed directional behavior (Fig. 4A). Once pacing was initiated, a change in the distribution was noted with a buffer period of about 15 min (Fig. 4B) before observing uniform entrainment of the slow wave in a single direction (Fig. 4C).

**Fig. 5:**
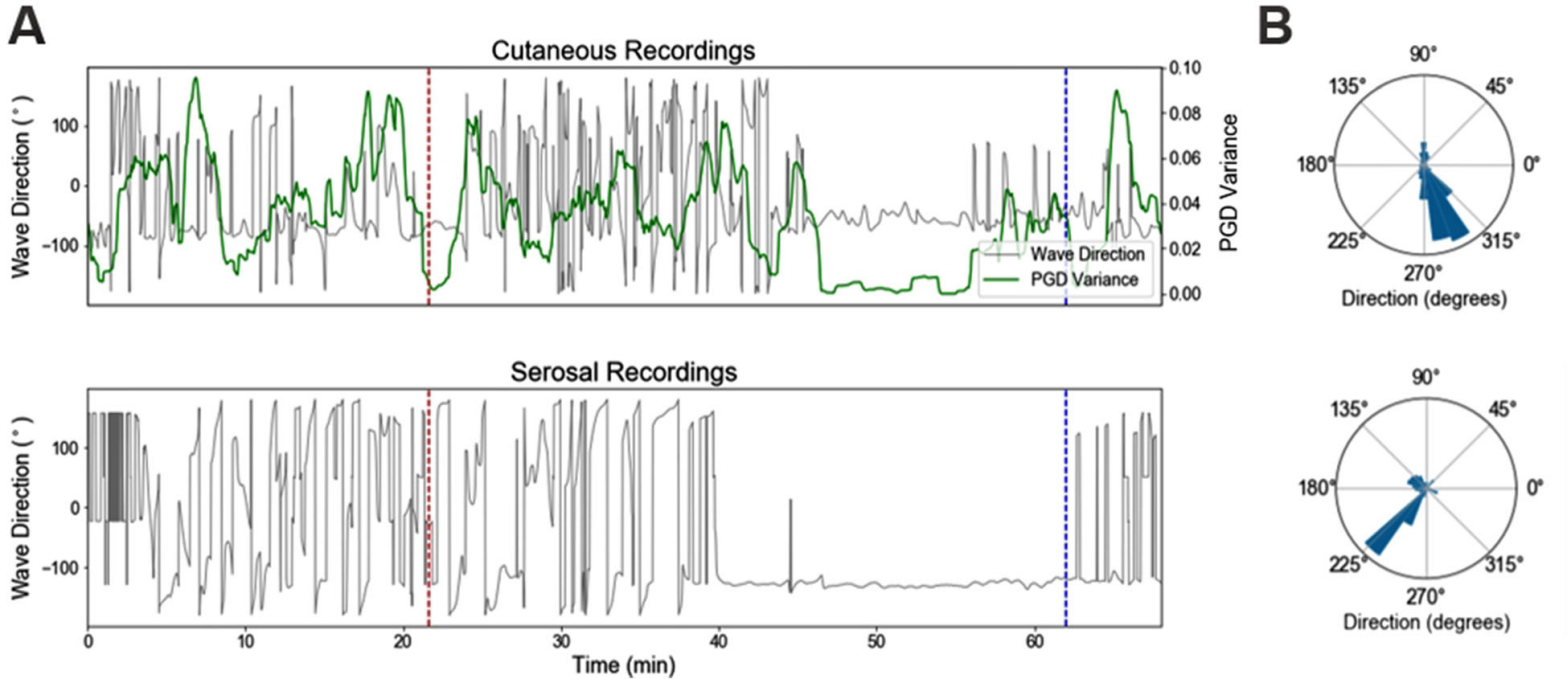
Slow Wave Direction over Time. **A** Slow wave direction is plotted over time in the cutaneous and serosal electrodes. Red dotted vertical line represents the start of pacing and blue dotted vertical line represents the end of pacing. A buffer period of about 15 min is observed prior to the formation of a constant direction in the slow wave. This constant direction is observed to continue until termination of the stimulus. As expected, PGD variance was noted to decrease during this period. **B** Spatial histogram of the direction of the slow wave is plotted for PGD > 0.5, demonstrating the formation of this uniform direction

This behavior was also observed in a direction plot of both the cutaneous and serosal electrode recordings (Fig. 5). While the stimulus was delivered at t = 22 min, the slow wave began propagating in a constant direction at t = 38 min. The PGD variance was plotted against time (Fig. 5A, green line) and utilized to generate spatial histograms of overall slow wave direction at time points for which PGD was > 0.5. Notably, the serosal and the cutaneous electrodes were analyzed separately but both found to demonstrate the same spatial and spectral behaviors in gastric electrical activity during manipulation by pacing. A 45-degree angle difference was observed between the recordings, likely due to differing 2D Cartesian frames of reference with the coordinates of each electrode array.

**Fig. 6:**
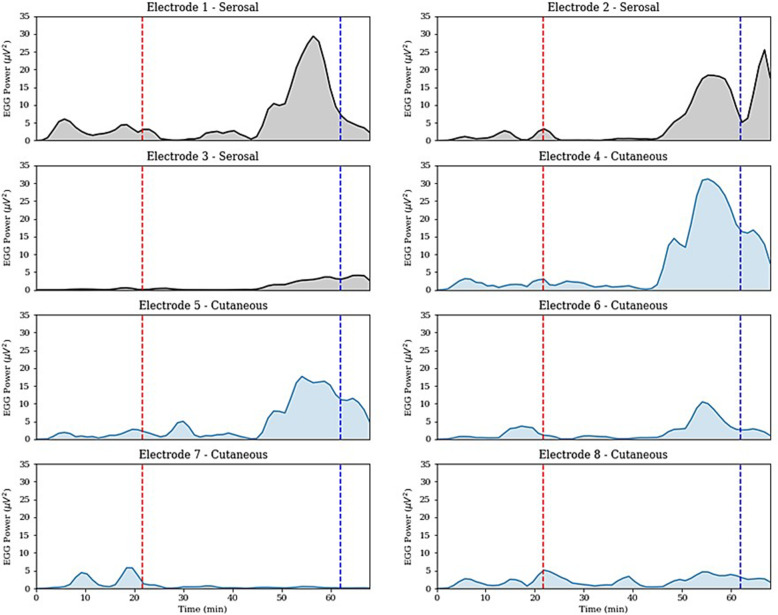
EGG Average Power. The average power in six of the eight electrodes was shown to increase during the same theorized entrainment period. A buffer period is also present in this analysis, where pacing has started but motor activity has not yet unified. Red dotted vertical line represents the start of pacing and blue dotted vertical line represents the end of pacing. Grey plots represent serosal electrode measurements, blue plots represent cutaneous electrode measurements

Further analysis showed that the average power in the EGG frequency band also spiked during the same time period that a consistent and coherent direction was observed (Fig. 6), suggesting that pacing-induced entrainment also produced stronger slow waves. Of note, two of the five electrodes had low overall signal strength, likely due to distant placement from the stomach, thus also missing the signal amplification during the entrainment period (a consequence of the inverse-square law).

This work presents a unique opportunity to monitor gastric activity in response to artificial pacing. This is not only pivotal for a wireless telemetry-based feedback control system, but it also serves as a means to dynamically track the physiological effects of gastric pacing. While many clinical studies have attempted to utilize ES as a means to treat gastric dysmotility (Abell et al. 2002; McCallum et al. 2011; McCallum et al. 2010; Forster et al. 2001; Lin et al. 2004; Mason et al. 2005; Levinthal and Bielefeldt 2017), there is still an incomplete understanding of the mechanisms of action by which this stimulation enhances motor function in the stomach. For instance, in the gastroparesis clinical trials, although patients were shown to have reduced symptoms of nausea and vomiting, there was little impact on gastric emptying (Abell et al. 2002; McCallum et al. 2011; Lin et al. 2004; Abell et al. 2003), a phenomenon which would be expected as a result of resolving gastroparetic dysmotility. The combination of cutaneous EGG and minimally-invasive wireless pacing allows for real time feedback regarding both the spectral and spatial effects of stimulation-based therapy while reducing the surgical footprint. Our prior work has demonstrated that spatial patterns from cutaneous multi-electrode EGG recordings correlate with severity of symptoms in patients with gastroparesis and functional dyspepsia (Gharibans et al. 2019). These findings thus provide a unique opportunity for an objective measure related to symptomatic improvement in a closed feedback loop with gastric pacing. Furthermore, this combined approach opens doors to other applications for pacing of the stomach. Notably, there have been various studies on the treatment of obesity using ES at different anatomical locations in the stomach (Bohdjalian et al. 2006; Peles et al. 2003; Chiu and Soffer 2015; Horbach et al. 2015), but targeted therapy has not yet been proven successful due to inconclusive results and a lack of complete understanding of the effects of electrical stimulation.

In this work, we demonstrated the successful entrainment of the gastric smooth muscle following a period of pacing. Notably, our cutaneous EGG recording system was able to capture these effects despite low spatial resolution measurements using only five cutaneous electrodes. While future studies will focus on higher resolution cutaneous recording, this work presents the fundamental capability of a cutaneous array of EGG electrodes to provide sufficient information for programmable therapeutics without the need for an additional internal recording implant. Furthermore, our pacing system has been designed for minimally invasive delivery and fixation via stent-like vascular implantation (Abiri et al. 2021). While additional studies on the optimized position for pacing are underway, potential pathways for delivery can include the left gastroepiploic or splenic veins (average 3.2 mm (Moody and Poon 1992) and 3.6 mm in diameter (Kurol and Forsberg 1986), respectively), avoiding arterial complications while reducing surgical footprint. While dislodgement and occlusion may occur after device implant, venous deployment can reduce the risk of adverse outcomes in the setting of device failure and replacement as it avoids critical arterial anatomy. As such, a wireless intravascular gastric pacemaker can significantly improve quality of life for patients due to reduced complications associated with device leads, including strangulation of the enteric system through lead entanglement that causes obstruction and necrosis, perforation of the stomach wall due to serosal helix fixation, and dislodgement simply due to patient movement or weight changes (Enterra 3116 Implant Manual 2015).

It is important to note the long road to clinical applicability. Future studies will focus on the impact of long-term pacing on gastric motility at different anatomical locations in the stomach. Targeted therapy can be established with an optimized device implant location. Using our previous work as a basis (Allegra et al. 2019), we can potentially target placement of the pacing electrodes using cutaneous multi-electrode arrays to perform source localization to pinpoint the region of the stomach for which aberrant slow wave activity occurs. This cutaneous system would then be utilized post-implant for feedback-based stimulus control. The closed-loop system could act in real-time when the slow wave direction or amplitude is outside the expected range, either increasing or decreasing stimulus strength by means of proportional integral derivative (PID) or state-space control with error from desired state as the input. Since the EGG signal is slow, we can also use a fuzzy control system appropriate for this task. Furthermore, the effect of chronic implantation on electrical insulation will need to be addressed with the investigation of biomaterials for long-term device encapsulation, including glass encapsulation (Vest et al. 2017) or flexible silicone rubber and oil-based parylene packaging (Shapero and Tai 2018).

Finally, directionality of pacing can impact gastric activity, with the ability to potentially wirelessly control transient dysregulation for applications in obesity and weight loss. Cutaneous EGG readings in combination with other imaging modalities may also help distinguish the basis for changes in gastric function due to pacing, including the combined roles of the motor, neural, and vascular systems. Recent studies have shown the impact that disturbed neurotransmission can have on disrupted slow wave activity (Klein et al. 2013), thus opening a potential role for pacing to override dysrhythmic nervous activity and facilitate a more anterograde peristalsis mechanism. Additional studies are warranted to better answer these questions. In this work, our analysis was based on the investigation of only a single animal. While the prolonged entrainment period in our data lends confidence in the results of our study, we note the importance of adjunct models to obtain a better understanding of gastric pacing in response to a stimulus, the time needed for entrainment, and the achievement of persistent entrainment post-stimulation.

## Conclusions

This work establishes the foundation for a miniaturized intravascular wireless pacer with a cutaneous array of EGG electrodes to enable closed-loop targeted gastric therapy. Our preliminary data demonstrates the ability to induce directional entrainment in the stomach smooth muscle using transient pacing. While additional studies are necessary for a better understanding of the mechanism of action of the entrainment and its sustainability, this work presents a basis for the feasibility of using a closed loop system of a wireless pacemaker and non-invasive array of recording electrodes as a means to treat gastric dysmotility.

## Data Availability

Not applicable.

## Abbreviations

ES: Electrical stimulation
EGG: Electrogastrography
SAR: Specific absorption rate
PGD: Phase gradient directionality
PID: Proportional integral derivative
